# Differential Biodegradation Kinetics of Collagen Membranes for Bone Regeneration

**DOI:** 10.3390/polym12061290

**Published:** 2020-06-04

**Authors:** Manuel Toledano, Samara Asady, Manuel Toledano-Osorio, Franklin García-Godoy, María-Angeles Serrera-Figallo, José A. Benítez-García, Raquel Osorio

**Affiliations:** 1 Faculty of Dentistry, Dental Materials Section, University of Granada, Colegio Máximo de Cartuja s/n, 18071 Granada, Spain; toledano@ugr.es (M.T.); samara_asady@hotmail.com (S.A.); info@drpepebenitez.com (J.A.B.-G.); rosorio@ugr.es (R.O.); 2 College of Dentistry, Health Science Center, University of Tennessee, 875 Union Avenue, Memphis, TN 381632110, USA; fgarciagodoy@gmail.com; 3 Faculty of Dentistry, Oral Surgery Section, University of Sevilla, Avicena s/n, 41009 Sevilla, Spain; maserrera@us.es

**Keywords:** collagen, membrane, biodegradation, bone regeneration

## Abstract

Native collagen-based membranes are used to guide bone regeneration; but due to their rapid biodegradation, this treatment is often unpredictable. The purpose of this study was to investigate the biodegradability of natural collagen membranes. Three non-cross-linked resorbable collagen barrier membranes were tested: Derma Fina (porcine dermis), Evolution Standard (equine pericardium) and Duo-Teck (equine lyophilized collagen felt). 10 × 10 mm^2^ pieces of membranes were submitted to three different degradation procedures: (1) hydrolytic degradation in phosphate buffer solution, (2) enzyme resistance, using a 0.25% porcine trypsin solution, and (3) bacterial (*Clostridium histolyticum*) collagenase resistance test. Weight measurements were performed with an analytic microbalance. Thickness was measured with a digital caliper. Membranes were analyzed at different time-points, up to 21 d of immersion. A stereomicroscope was used to obtain membranes’ images. ANOVA and Student Newman Keuls were used for mean comparisons (*p* < 0.05), except when analyzing differences between time-points within the same membrane and solution where pair-wise comparisons were applied (*p* < 0.001). Derma Fina attained the highest resistance to all degradation challenges. Duo-Teck was the most susceptible membrane to degradation, complete degradation occurred as soon as 8 h. The bacterial collagenase solution performed as the most aggressive test as all membranes presented 100% degradation before 21 d.

## 1. Introduction

The aging population is the principal reason to advance the understanding of dental biomaterials aimed to achieve periodontal regeneration [1]. Chronic periodontitis is one of the most aggressive pathologies and it refers to the inflammation and progressive destruction of the supporting tissues of the periodontium resulting in loss of the teeth and the alveolar bone. Restoration of the lost periodontium is a paramount goal of periodontal therapy [2], where the guide bone regeneration (GBR) concept plays a capital roll. GBR is a surgical procedure that uses membranes as barriers to prevent the ingrowth of fibroblasts and to maintain a space for osteogenesis. The use of a barrier membrane to promote the selective repopulation of a periodontal/bone defect by cells with regenerative potential has been successfully applied for more than 40 years when the first application of a cellulose acetate laboratory filter by Millipore for GBR occurred [3]. Currently, GBR is a method successfully employed in dental practices aimed to increase the volume of the host bone at sites with insufficient bone quantity [4].

Two main types of barrier membranes are available on the market: resorbable and non-resorbable. Non-resorbable membranes (as polytetrafluoroethylene -PTFE-) maintain their structural integrity for as long as they are left in the tissue, providing the operator with complete control over the time of application [5]. They also guarantee space maintenance for the duration of healing, but require a second surgery for their removal. Complications have also been reported such as frequent soft tissue dehiscences or infections during membrane exposure, which can negatively influence clinical outcomes or regenerative procedures, jeopardizing osteogenesis [6,7,8]. The resorbable membranes are those composed by synthetic polymers like polylactic or polyglycolic acid or by biopolymers like collagen [3]. Collagen membranes are the most frequently employed, and the ones with the highest number of reported clinical studies available [3]. Collagen membranes exhibit several advantages, compared to synthetic polymeric membranes, such as easy manipulation, weak immunogenicity, a direct effect on bone formation and chemotaxis of gingival and periodontal ligament fibroblasts. Some other benefits include promoting wound healing and stability through isolation, clot stabilization, hemostasis, semi-permeability, support of nutrient transfer and augmenting flap thickness by providing a collagenous scaffold [9,10,11]. The source of the collagen varies, but it is typically obtained from bovine tendon, bovine dermis, calf skin or porcine dermis [12]. Porcine skin-derived collagen membranes are widely used in GBR procedures because porcine dermis primarily consists of type I collagen and its 3D architecture is similar to that of native extracellular matrix [13]. Purified bovine and porcine collagen derived from tendons, dermis (skin), pericardium and other regions has also been shown to be suitable as donor material [1]. However, the major drawback of native collagen is the rapid biodegradation by the enzymatic activity of macrophages and polymorphonuclear leucocytes. Then, the potential of losing space maintenance ability in physiological conditions is high [3]. If the membrane dissolves quickly, clinical treatment goals will not be achieved and GBR will be unpredictable [13]. An ideal barrier for GBR should resorb gradually over time [9]. Although the biodegradable nature of biological membranes eliminates the need for surgical membrane retrieval, these collagen membranes present limitations in terms of controlling degradation [2]. It has been suggested that these membranes must stay physically and mechanically intact for at least 4–6 weeks for regenerative therapy to be successful [2]. Therefore, unsuitable degradation rates may significantly hamper the regenerative potential of currently available GBR membranes [14]. 

Only a limited number of studies have investigated the resorption patterns of collagen membranes, showing that their degradation might start within 4 days to 6 weeks after surgical placement. However, in most of the published studies, resorption of collagen membrane was evaluated in subcutaneous tissue membranes implantation in rats. Moreover, the available data mainly consist of qualitative histologic observations and/or the measurement of membrane thickness, with little characterization of the enzymatic or hydrolytic degradation process [9]. In vitro findings suggest that the composition and/or structure of the membrane may play an important role in the clinical outcomes of degradation. The activity of bacteria and their enzymes in sites of regeneration may contribute to the rapid elimination of membrane material and shorten the desired period of regeneration considerably [15]. 

Resorption time of collagen membranes may be extended by cross-linking of the fibers through physical or chemical procedures. These cross-linked collagen membranes are not usually employed clinically, because it has been shown that cross-linking using glutaraldehyde decreased the biocompatibility whereas enzymatic cross-linking negatively reduced the tissue integration and biodegradation pattern. The degree of chemical cross-linking caused severe inflammatory reactions [11,16]. Even more, one of the questions is whether non cross-linked membranes really display differences in terms of degradation behavior. Therefore, non-crosslinked collagen membranes were selected for the present study.

Absorbable barrier membranes offer limited control over the length of application because their inherent nature allows the disintegration process to start upon placement in the tissue. Porosity is an important feature of membranes as they allow for the infiltration of nutrients into the defect, which promotes bone growth [17]; however, the excessively large pores after biodegradation might make the membranes less effective as a barrier against soft tissue cells. Provided that the longevity of the barriers’ function is an important aspect of their clinical performance, the loss of the structural integrity of collagen membranes due to fast biodegradation by enzymatic activities becomes a major problem of this type of bioabsorbable devices [5]. The membrane thickness and weight affect the mechanical properties of the membrane and define the diffusion distance between tissue compartments and, therefore, provide a rationale for selecting membranes in view of specific applications in GBR [18]. Then, investigations on variations in these properties or in the possibility of microstructural membranes defects formation during their degradation processes is a crucial point to be considered in order to increase the success of GBR therapy. The necessity and novelty of the present study is justified.

Hence, the aim of this study was to investigate the degradation kinetic, from 4 h to 21 d, of three non-crosslinked collagen membranes from different origin. A qualitative microstructural assessment and a quantitative analysis of the collagen membrane degradation were performed. The null hypotheses to be tested were that: (i) the three membranes for guided bone regeneration do not degrade in the same extent, over time; and (ii) the three membranes do not resist similarly the different degradation processes (hydrolytic, bacterial collagenase and trypsin).

## 2. Materials and Methods

### 2.1. Membranes Tested

Three GBR collagen membranes were tested. Membranes are commercially available and CE-certified for oral applications and all have heterologous origin. The membranes tested were: (1) Derma Fina (OsteoBiol^®^ by Tecnoss, Torino, Italy); (2) Evolution Standard (OsteoBiol ^®^ by Tecnoss, Torino, Italy); (3) Duo-Teck (OsteoBiol ^®^ by Tecnoss, Torino, ^®^ by Tecnoss, Torino, Italy). According to the manufacturer, Derma Fina is derived from porcine dermis after removal of the epithelial layer. The membrane is composed of a network of highly purified non-cross-linked porcine collagen fibers intermingled with porcine elastin fibers. This membrane is made of non-cross-linked porcine Type I and III collagens and has a bi-layered structure. The processing technique is performed at room temperature (cold process). Evolution Standard is a resorbable dense collagen mesh barrier derived from heterologous mesenchymal equine pericardium tissue, and Duo-Teck is a resorbable membrane derived from equine lyophilized collagen felt, one of the external surfaces is covered by micronized equine bone particles (up to 300 µm).

### 2.2. Degradation Assays

Membrane samples were cut to a size of 10 × 10 mm^2^. Three specimens of each membrane type were employed for each test and further measured in weight and thickness. For weight (W) measurements, an analytic scale (A&D-Instruments, Frankfurt, Germany) was used, with an accuracy of 0.0001 g; the complete device was mounted on an antivibratory table. Thickness (Th) was measured at random positions by means of a digital caliper (Mitutoyo 293-561, Tokyo, Japan). Three different degradation tests were performed: 

(1) *Hydrolytic degradation test:* In vitro hydrolytic degradation behavior of membranes was analyzed in phosphate buffer solution (PBS) at 37 °C [19].

(2) *Enzyme resistance test:* Samples were immersed in a 0.13% porcine trypsin solution (Sigma-Aldrich, St. Louis, MO, USA), and incubated at 37 °C [13]. 

(3) *Bacterial collagenase resistance test:* A collagenase solution from *Clostridium histolyticum* bacteria Type V (Sigma Aldrich, St Louis, MO, USA) was used. It is actually a mixture of several different enzymes including collagenase, which act together to break down tissue. This preparation contains collagenase, non-specific proteases, clostripain, neutral protease, and aminopeptidase activities. Specific activity is ≥125 CDU/mg solid. A collagenase concentration of 2 IU/mL in 50 mM Tris HCl (pH 7.4) containing 10 mM CaCl_2_ was used [15,16]. After each 48 h, degradation solutions were removed carefully through suction and renewed [2]. After each immersion time point (4 h, 8 h, 12 h, 16 h, 24 h, 48 h, 72 h, 7 d, 14 d, and 21 d), samples were dried by placing them into a vacuum chamber at 37 °C for 72 h. Next, the weight and thickness of the dried samples were measured. Three measurements were taken from each specimen. All degradation experiments were performed in triplicate. At the end of the storage, pictures of the membranes’ surfaces were obtained by means of an Olympus SZ-CTV stereomicroscope (Olympus, Tokyo, Japan) a digital signal processor DSP 5050 (Olympus, Tokyo, Japan) was used.

### 2.3. Statistical Analysis

Multiple ANOVA models were used to assess the influence of the independent variables (degradation solution, type of membrane and immersion time) on the dependent variables (weight and thickness). Analyses of interactions were also performed. ANOVA and Student Newman Keuls post-hoc comparisons were performed to determine differences between materials and degradation solutions. To permit for these comparisons, the variables weight and thickness were converted to percentage of variation respect to the initial measurement following the equation:

Percentage of loss = [(X_0_ − X_t_)/X_0_] × 100, (1)

where, X_0_ is the initial weight or thickness of specimens; and X_t_ is the specimen’s weight or thickness at each time-point (t). 

Pairwise comparisons were performed to ascertain for differences between immersion time-points within the same membrane and solution experimental group. Normal distribution of data was probed before the analyses and statistical significance was always considered at *p* < 0.05 except for pairwise comparisons where a Bonferroni’s correction was applied and *p* < 0.001 was set. Statistical analysis was performed using SPSS 25.0 (SPSS Inc., Chicago, IL, USA) software package. 

### 2.4. Light Microscopy Analysis

Before immersion and at the end of the storage period (21 d), specimens were observed under a stereomicroscope Olympus SZ-60 (Olympus, Tokyo, Japan) for microstructural analysis. Images were taken at 60× and 120× magnifications.

## 3. Results

**Figure 1:**
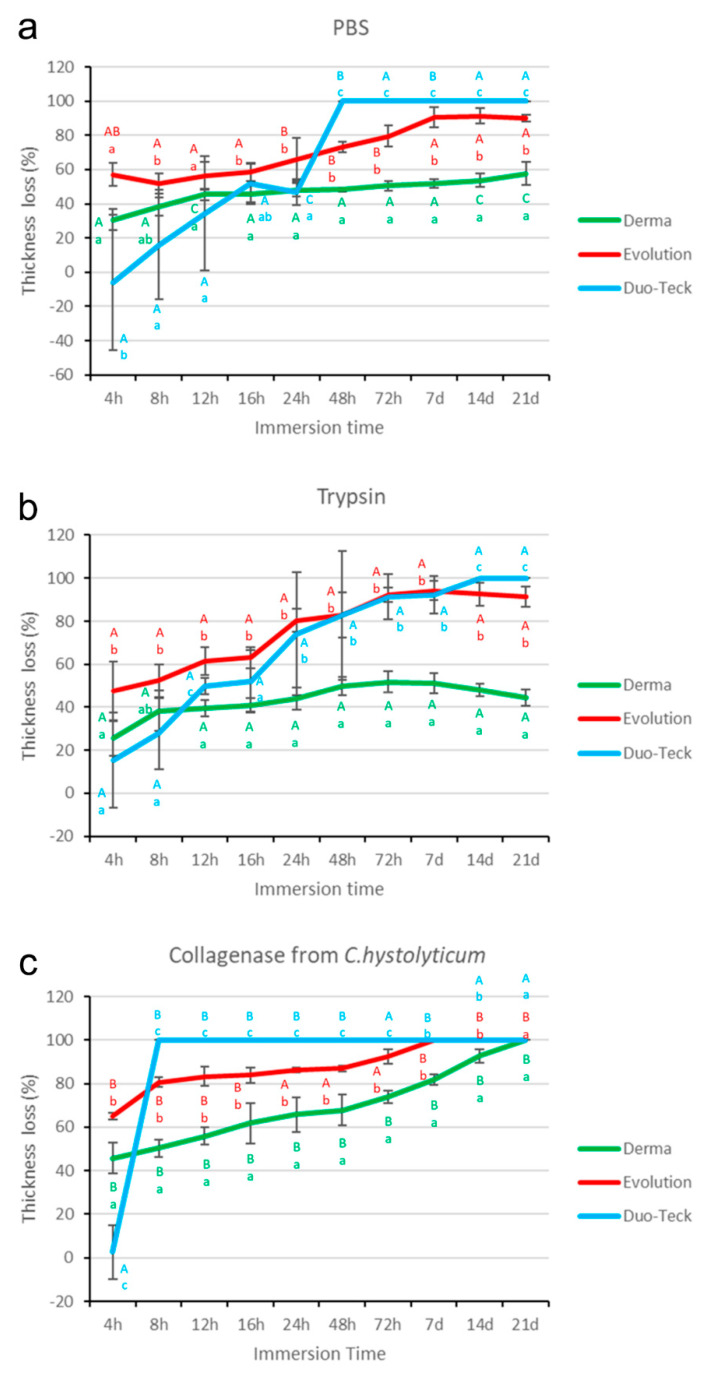
Degradation profile by % thickness loss analysis of the three experimental membranes (Derma Fina, Evolution Standard and Duo-Teck) over immersions periods up to 21 days in: (**a**) PBS, (**b**) trypsin and (**c**) collagenase from *Clostridium histolyticum*. Values shown are mean and standard deviation (*n* = 3). Significant differences between membranes within the same immersion solution are noted by low-case letters. Differences between different immersion solutions within the same membrane are pointed out with capital letters. Multiple comparisons were performed by Student-Newman-Keuls (*p* < 0.05).

The thickness (Th) values in mm of the three membranes (Derma Fina, Evolution Standard and Duo-Teck) submitted to the three different degradation tests (PBS, trypsin and *C. histolyticum* collagenase) as a function of the different time-points are reflected in Table 1. The loss of percentage thickness (Th) values of the membranes, as a function of the different degradation tests and time-points are represented in Figure 1.

### 3.1. Thickness Evaluation after PBS Degradation Assay

At 4 h time-point, Duo-Teck attained lower loss of Th values tan both Derma Fina and Evolution Standard, which performed similarly. The trend was as follows Derma Fina = Evolution Standard > Duo-Teck. After 12 h of storage, the three membranes performed similar with the trend Derma Fina = Evolution Standard = Duo-Teck. After 16 h of storage, Derma Fina attained the lowest and Evolution Standard the highest loss of Th values, respectively, and Duo-Teck reached an intermediate performance between both. The trend was as follows Evolution Standard ≥ Duo-Teck ≥ Derma Fina (Figure 1a). After 48 h and 72 h of storage, the highest loss of Th was attained by Duo-Teck which totally degraded. Derma Fina showed the lowest loss of Th values. The trend was as follows: Duo-Teck > Evolution Standard > Derma Fina. At 7, 14 and 21 d all samples followed the same trend: i.e., Duo-Teck > Evolution Standard > Derma Fina (Figure 1a).

In general terms, Duo-Teck totally degraded after 48 h of storage. Both Derma Fina and Evolution Standard membranes had an ascending and parallel loss of thickness over time, but differentiated from 24 h until 21 d. The loss of Th in the three membranes was significantly increasing according to the different time-points of the study when compared with the initial thickness, except Duo-Teck that attained significant differences after 12 h of storage (Table 1).

### 3.2. Thickness Evaluation after Trypsin Degradation Assay

At 4 h time-point, both Derma Fina and Duo-Teck membranes reduced their percentage thickness (Th) similarly, whose values were lower than those of Evolution Standard. Similar performance was followed after 16 h and 7 d of storage, but with different percentages values (Figure 1b). At 14 d time point, the membranes performed as follows respect to the loss of Th: Duo-Teck > Evolution Standard > Derma Fina. This outcome indicated that Duo-Teck completely disappeared after immersion in trypsin degradation solution. Similar performance was followed at 21 d of storage, with a similar percentage Th loss (Figure 1b).

In general terms, both Evolution Standard and Duo-Teck membranes suffered and ascending loss of Th over time, more accentuated in Duo-Teck, which totally disappeared at 14 d time-point. As early as 16 h of storage, Evolution Standard attained significant differences over time, when all time-points were compared with the initial time. Duo-Teck started to loose thickness after 24 h time-point until the end of the study (Table 1). Degradation of Derma Fina reproduced a mild parabolic track in degradation. From 8 h until 21 d, the loss Th values were significant when compared with the initial time (Table 1).

### 3.3. Thickness Evaluation after C. histolyticum Collagenase Degradation Assay

At 4 h of storage, the membranes performed as it follows respect to the loss of Th: Evolution Standard > Duo-Teck > Derma Fina (Figure 1c). After 7 d of storage, both Evolution Standard and Duo-Teck completely degraded as the loss of Th was complete at both. Derma Fina showed the lowest loose of Th values and the trend was as follows: Derma Fina < Evolution Standard = Duo-Teck. Similar performance was followed at 14 d time-point but with different percentage. At 21 d time-point, all membranes completely degraded as the loss of Th was complete for the three membranes (Figure 1c). 

In general, Duo-Teck totally degraded after 8 h of storage. Both Derma Fina and Evolution Standard membranes had a parallel degradation over time, but Evolution Standard disappeared after 7 d and Derma Fina at 21 d time-points (Table 1). The loss of Th in the three membranes was significantly increasing at the different time-points of the study, when compared with the initial thickness, except Duo-Teck thickness loss that attained significance at 4 h of storage (Table 1).

**Figure 2:**
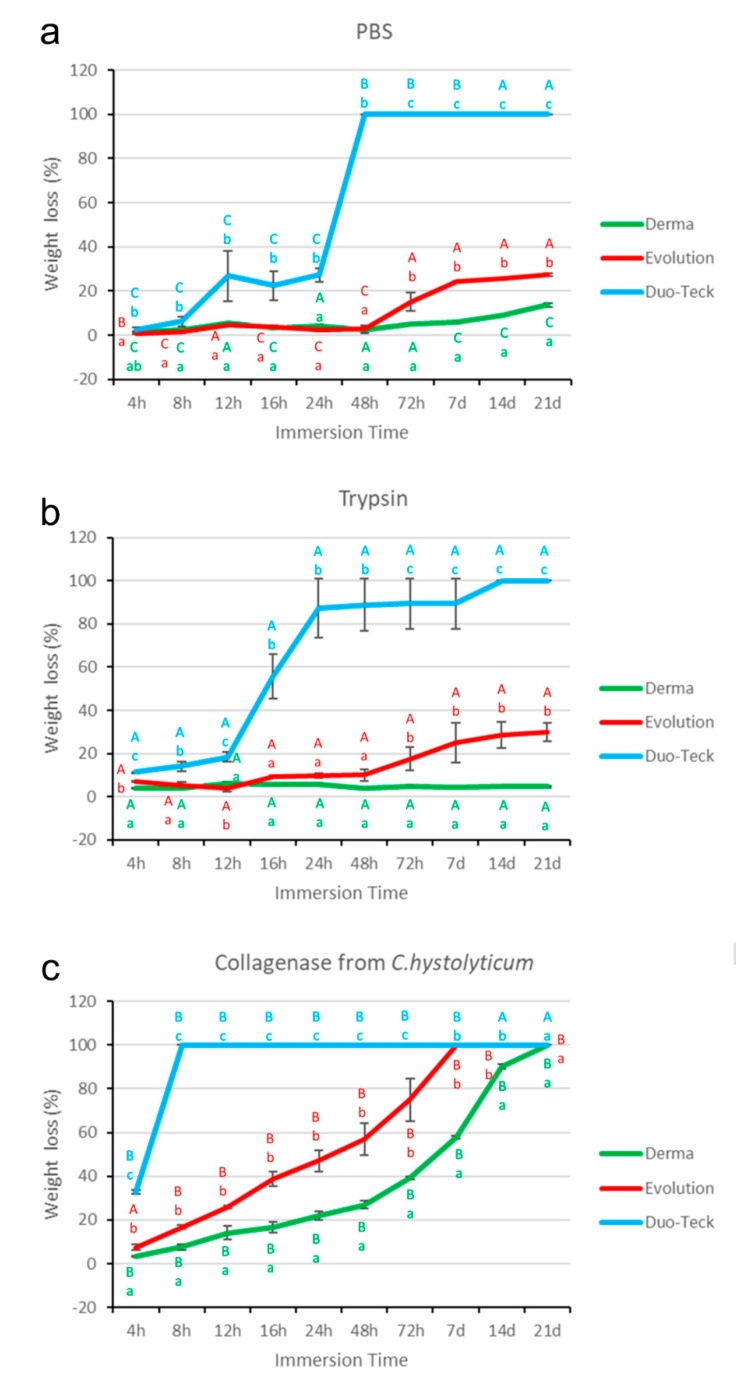
Degradation profile by % weight loss analysis of the three experimental membranes (Derma Fina, Evolution Standard and Duo-Teck) over immersion periods up to 21 days in: (**a**) PBS, (**b**) trypsin and (**c**) collagenase from *Clostridium histolyticum*. Values shown are mean and standard deviation (n = 3). Significant differences between membranes within the same immersion solution are noted by low-case letters. Differences between different immersion solutions within the same membrane are pointed out with capital letters. Multiple comparisons were performed by Student-Newman-Keuls *p* < 0.05).

The weight (W) values in g of the three membranes (Derma Fina, Evolution Standard and Duo Teck) submitted to the three different degradation tests (PBS, trypsin and *C. histolyticum* collagenase) as a function of immersion time are reflected in Table 2 and Figure 2.

### 3.4. Weight Evaluation after PBS Degradation Assay

At 4 h time point, Evolution Standard attained the lowest loss of W, and Duo-Teck the highest loss of W values. Derma Fina showed an intermediate value and no differences were found between them. The trend was as follows: Duo-Teck ≥ Evolution Standard = Derma Fina (Figure 2a). At 72 h time point, Duo-Teck was completely degraded and Derma Fina obtained the lowest loss of W, with the trend: Derma Fina < Evolution Standard < Duo-Teck. Similar performance was achieved at 7 d, 14 d and 21 d time-points but with different W loss percentages (Figure 2a).

In general terms, at 24 h time-point, Duo-Teck reduced is W approximately and at 48 h of storage which indicates a complete degradation. Both Derma Fina and Evolution Standard presented lower loss of W after 72 h of storage, and significant differences existed between the two membranes which remained until the end of the study (Figure 2a). The loss of W in the three membranes was significantly increasing according to the different time-points of the study when compared with the initial time (Table 2).

### 3.5. Weight Evaluation after Trypsin Degradation Assay 

At 4 h time-point, Duo-Teck showed the highest and Derma Fina the lowest loss of percentage weight, with the trend: Duo-Teck > Evolution Standard > Derma Fina (Figure 2c). Similar performance was followed at 12 h, 72 h, 7 d, 14 d and 21 d (Table 2) (Figure 2b).

In general terms, at 12 h time-point, Duo-Teck reduced is W at 24 h of storage. This loss of W stabilized until 7 d and totally degraded at 14 d of immersion. Both Derma Fina and Evolution Standard presented similar lower loss of W until 72 h of storage, where significant differences appeared between the two membranes which remained until the end of the study (Table 2).

### 3.6. Weight Evaluation after C. histolyticum Collagenase Degradation Assay 

At 4 h time-point, Duo-Teck attained the highest and Derma Fina the lowest loss of percentage W, with the trend: Duo-Teck > Evolution Standard > Derma Fina. At 7 d time point, both Evolution Standard and Duo-Teck membranes completely degraded. At 21 d time point, a complete degradation was attained for all of them (Figure 2c).

In general terms, Duo-Teck totally degraded after 8 h of storage. Both Derma Fina and Evolution Standard membranes had a parallel degradation kinetic behavior over time, but Evolution Standard completely degraded after 7 d and Derma Fina resisted 21 d time points (Table 2). 

### 3.7. Membranes Morphological Analysis 

**Figure 3:**
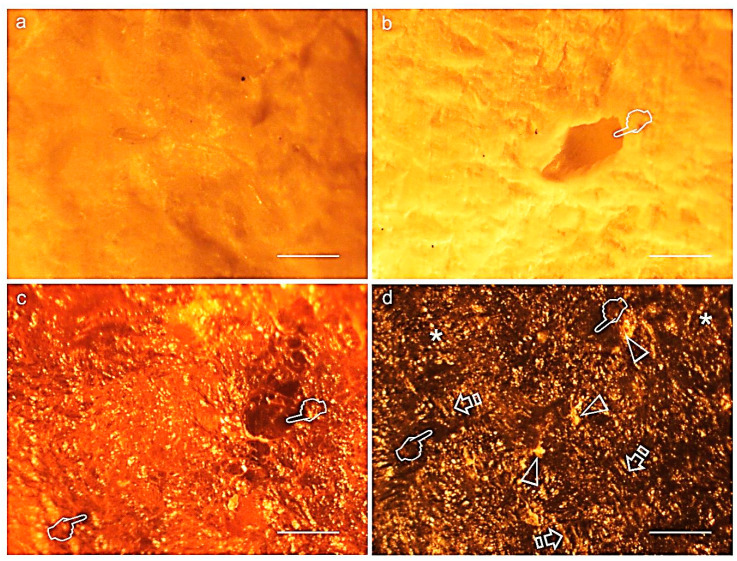
Representative light micrographs of surfaces of Derma Fina membrane at the outer (**a**), inner (**b**) surfaces before immersion and at the inner surfaces after trypsin (**c**) and PBS (**d**) immersion, at 21 d of storage. Single arrows indicate bundles of collagen. Both membrane faces (**b**,**c**) showed the typical crumpled appearance of immersed membranes. Large diameter pores (wider than 100 µm) were observed at the surface of the membrane (pointers). Shorter and thinner fibers were seen after 21 d in trypsin, and large and thicker fibers characterized the membrane surface after biodegradation in PBS (asterisks). A compact collagen arrangement was interrupted by clusters of circular discontinuities, probably mineral depositions (arrow heads). Scale bar is 1000 µm in (**a**,**c**,**d**); and 2000 µm in (**b**).

**Figure 4:**
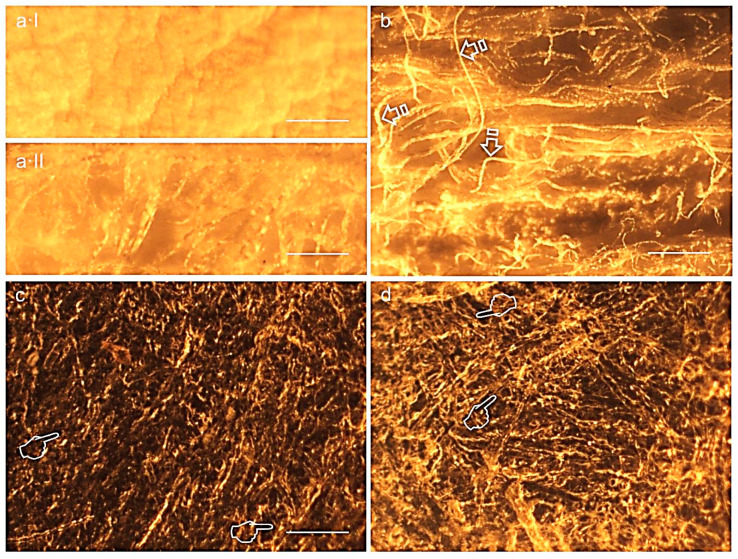
Representative light micrographs of surfaces of Evolution standard membrane at the outer (**a**·I), inner (**a**·II, **b**) surfaces before immersion and at the inner surfaces after trypsin (**c**) and PBS (**d**) immersion, at 21 d of storage. The ondulating collagen fibers and microfibrils were arranged in stacked layers, which were parallel to the membrane surface (single arrows). Collagen fibrils formed fine, loosely arranged, undulating collagen bundles. The packing of fibrils into fibers was not very tight, resulting in single fibrils or small bundles interconnecting larger bundles. Isolated fibrils running crosswise formed a loose meshwork overlying the bundled fibrils. Multiple grooves, pits and pores (pointers) were seen at the inner surface of the membrane. Scale bar is 1000 µm in (**a**·I), (**a**·II), (**c**), (**d**) and 2000 µm in (**b**).

Figure 3 and Figure 4 display light micrographs taken from membranes after degradation testing. Both Derma Fina and Evolution Standard membranes displayed a smooth and compact surface morphology on the outer surfaces (Figure 3a and Figure 4a·I). The inner surfaces were fibrous and irregularly crumpled (Figure 3b and Figure 4a·II). As seen from the rough side (Figure 3c,d), the different collagen layers consisted of reticulated fibers. Multiple bundles of collagen of different sizes were aligned in parallel and were separated from each other by irregular spaces when Derma Fina was immersed in PBS (Figure 3d). In this membrane, the collagen fibers were also arranged in layers, which were paralleled to the surface. Within these strata, the degree of collagen fiber orientation was variable. While some layers of collagen fibers typically showed less uniformity regarding collagen fiber orientation and thus resembled an interwoven fiber network, others exhibited a higher degree of preferential fiber orientation. 

**Figure 5:**
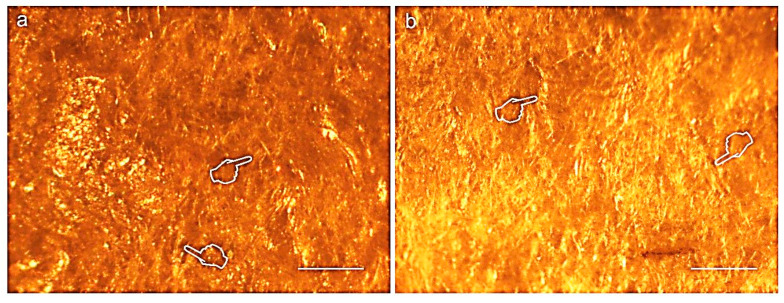
Representative light micrographs of surfaces of Duo-Teck membrane at the outer (**a**) and inner (**b**) surfaces before immersion. The membrane has a surface formed by numerous frames arranged in a disordered manner, with large gaps intermingling the entire structure (pointers). Scale bar is 1000 µm in (**a**), and 2000 µm in (**b**).

Before immersion, the structural collagen pattern of Evolution Standard presented a profile of dense distribution of filaments with a compromise parallelism among fibers and fewer detectable pores (Figure 4b). Largely different morphologic features were not found when Evolution membranes were compared after 21 d of immersion in the two different media, trypsin vs. PBS solutions (Figure 4c,d). Optical microscopy clearly exhibited regions with larger size pores (wider than 100 µm) which were located in the same areas in which the collagen fibrils were absent. Residual collagen showed a particularly pronounced ripple in this membrane type when immersed in both trypsin and PBS solution. The collagen fibers were arranged in layers that paralleled the surface. The degree of preferential fiber orientation was variable (Figure 4c,d). Collagen fibers took a straight course and underwent extensive branching. These fibers were most abundant in the superficial tissue layers, but their occurrence remained noticeable in deeper layers as well (Figure 4d). Duo-Teck membrane had a surface arranged in nonhomogeneous layers, fibrous, fuzzy and irregular, interspersed with a multiple porous surface and also a wide variety of pore diameters at both sides of the membrane were found (Figure 5a,b).

## 4. Discussion

After assessing the results obtained in the present research, it may be assumed that Derma Fina GBR membrane demonstrated the greatest resistance to the different degradation challenges under in vitro conditions (Table 1 and Table 2; Figure 1 and Figure 2). 

Both Evolution Standard and Duo-Teck membranes have a similar composition that is primarily equine collagen, though collagen in Duo-Teck was in a lyophilized state. Duo-Teck was the membrane most susceptible to biodegradation, as both W and Th loss attained values of total degradation (100%) in the three tested immersion media. Membranes integrity was affected even from 4 h, where pronounced signs of degradation were adverted, in weight and thickness. These data confirms previous findings [9], a double layer porcine collagen membrane in vivo degradation assay showed a significant reduction in membrane thickness from 14 to 30 d of healing, as well as in the total amount of collagen. One possible reason for this phenomenon is that the lyophilizing treatment undertaken in Duo-Teck membrane might partially had denatured the 3D architecture of the membrane itself, causing the misalignment of side chains and leading to membrane brittleness. Another possible reason could be that the re-hydration and lyophilization procedure leads to loose connection between collagen fibers [13] making them more susceptible to the different degradation biological processes. Moreover, it is the only membrane exhibiting a slight increase in thickness after the first 4 h of immersion (negative values were attained at the % thickness loss assessment), which may be also due to conformational changes produced after hydration at the loosely connected collagen fibers. Anyhow, a fact that has to be also considered is that this type of atypical structural conformation of collagen which has shown this membrane (Figure 3a,b) has become to be more susceptible to degradation, reducing the time of bioabsorption, and diminishing its potential use as a physical barrier [20]. For this reason, the first null hypothesis had to be accepted.

Differences in collagen tissue structure lead to different biological responses and hence account for their specific potential in regenerative medicine [18]. Before immersion, optical microscopy images generally showed a non-porous structure at the upper or outer surface of both Derma Fina membranes (Figure 3a and Figure 4a·I). Both Derma Fina and Evolution Standard membranes were found to be less porous (Figure 4a) in comparison with other membranes of animal origin. A characteristic less porous membrane seems to be clinically accepted by the clinicians considering the need to avoid an excessive humidity and subsequent loss of the membrane´s physical properties [20]. The inner surface of Derma Fina was rough and possessed a porous structure with micro- as well as macropores (20–50 μm) (Figure 3b). On the contrary, the presence of multiple microfibrils and filaments characterized the inner surface of Evolution Standard membrane (Figure 4b). Microfibrils or subfibrils are filamentous subunits of the collagen fibril [18]. Derma Fina membrane is made of types I and III collagen. Native collagen types I and III have shown biocompatibility in vitro and in vivo, as well as fast vascularization and revitalization [11]. Derma Fina attained an increase of W loss of ~1.2 fold when trypsin was used as degradation media from 4 h to 21 d, and of ~7.6 fold when using PBS, over time. A loose meshwork of bundles fibrils were observed in the Derma Fina membrane stating the presence of large diameter pores (wider than 100 µm), and corroborating those findings (Figure 3c,d). The decrease percentage in Th values, in the same range of time, was of ~1.7 and 1.9, respectively in both degradation tests (Figure 1 and Figure 2), which permitted to observe thicker and larger collagen fibers (Figure 3d). Concerning Evolution Standard membrane, the decrease in W loss was of ~4.2 fold when trypsin was used as degradation test from 4 h to 21 d, and of ~30 fold when using PBS, over time. On the other hand, the decrease percentage in Th values, in the same range of time was of ~1.9 and 1.6 fold, respectively, in both degradation tests (Figure 1 and Figure 2). These comparisons denote the greater stability of Derma Fina when it was compared to Evolution Standard, they reflect a delay in the degradation behavior of the porcine-derived membrane, and they confirm that the degradation of collagen GBR membranes varies in relation to the tissue of origin [21]. The bilayer structure of Derma Fina may also have contributed to this chemical stability [9]. Our outcomes are in line with those found by Fickl et al., [22], who reported a slow resorption process of Derma Fina GBR membrane in beagle dogs after 4 months, almost preserving the complete original membrane thickness, indicating this biomaterial for space maintaining when treating recession type defects, as an alternative to subepithelial connective tissue grafts. In parallel, a native porcine types I and II collagen matrix revealed predictable results in enhancing the width of keratinized gingiva, reducing patient morbidity and operation time [11]. On the contrary, another porcine skin-derived collagen membrane (Bio-Guide) exhibit rapid degradation at 12 h of immersion in porcine trypsin solution (5% percent mass remaining) and was not visible after 24 h of immersion [13]. Bio-Guide, a natural biodegradable membrane (porcine I/III) unveiled a significant change in its thickness (~100 µm reduction at 3 weeks of immersion) between 4 and 8 weeks after implantation in experimental animals [13]. However, these results can only be used as a reference because the test was performed in a sterile environment and only one enzyme was evaluated on the different collagen membranes. Further research is required to assess whether the result obtained in the present in vitro study can be directly extrapolated to the clinical situation as it has been reported that *Porphyromonas gingivalis*, *Treponema denticola*, and *Bacteroides melaninogenicus* are capable of producing other collagenases, which can also result in premature degradation of GBR membranes [13].

The collagenase solution from *C. histolyticum* has become to be the most aggressive medium for membrane degradation, in the present research. At the end of the study (21 d), any membrane resisted against the *C. histolyticum* collagenase in vitro degradation. (Figure 1 and Figure 2). It must be noted that bacterial collagenase has been shown to be more effective in degrading collagen-based membranes, compared with the enzyme and the hydrolytic degradation assays. Therefore, this procedure can be considered as a worst-case scenario for studying the effect of exposure of collagen membranes to bio-resorption [16]. Microbial collagenases belong to a family of metalloproteinases (predicted to be Zn-dependent) obtained from *C. histolyticum* with collagenolytic activity. The activity of collagenase is specific on collagen and gelatine. The polypeptide chains of collagen are composed of numerous repetitions of tripeptide aminoacids sequence Gly-Pro-X, where X is often a proline, which post-translationally gets converted to hydroxyproline. The structure of collagenase from *C. histolyticum* occurs in two isoforms collagenase G (Col_G) and Col_H, multidomain proteins of ~115 KDa of gluzincin superfamily of metalloproteases [23]. Present in vitro results are in agreement with other in vivo studies. Hence, it has been demonstrated that native, non-cross-linked collagen had a fast tissue integration and vascularization paired with slight to no signs of ingrowing inflammatory cells. However, native and defatted types I/III collagens were almost completely resorbed after 12 weeks. This is in accordance to previous studies, which also found high biocompatibility and fast biodegradation for native, non-cross-linked collagens, i.e., after insertion into surgical pouches of mongrel dogs, where the specimens revealed severe to moderate degradation within 4–8 weeks [11]. But this relatively fast resorption could also be explained by the presence of periodontal pathogens such as *Porphyromonas gingivalis* and *Treponema denticola*. These pathogens were observed to produce collagenases and thus promote a premature degradation of collagen. However, these circumstances only applied in case of exposure to the oral cavity [11].

The biodegradation of the non-cross-linked Bio-Guide membrane has been evaluated in several studies, which challenged the manufacturers’ claim that the resorption of the membrane is first reported after 4 months. Slight to moderate degradation was observed already after 1 month in the oral cavity of dogs and severe to total degradation was observed at 4 m time-point [24]. Fragmentation and dissolution of membrane material implanted in subcutaneous pouches in rats was also reported following 3 weeks [25], and 3 weeks after implantation in rat calvaria bone [26] the membrane exhibited different resorption patterns [5]. Thus, Figure 1 and Figure 2 are shown that the samples subjected to *C. histolyticum* challenge lost more thickness and weight than those in trypsin and PBS alone. When samples were immersed in PBS, Derma Fina behaved as the membrane which was less effectively degraded after immersion, as ~42% of its thickness and ~86% of its weight remained in the solution, meanwhile ~10% of thickness and ~73% weight in Evolution Standard resisted degradation (Figure 1a and Figure 2a). At the end of the study (21 d), Derma Fina was the less affected membrane after trypsin immersion, as ~56% of its thickness and ~95% of its weight remained in the solution, meanwhile ~9% of thickness and ~70% weight in Evolution Standard resisted degradation (Figure 1b and Figure 2b). Nevertheless, these findings should be interpreted with caution as the results are expressed in percentages. The increase in number and size of pores after biodegradation of Evolution Standard made decrease the density of the material, compromising its function as a protective barrier against soft tissue cells [20] and hence producing a tentative detriment in the mechanical properties of the residual membrane [17]. Further research is required at this point.

To the best of our knowledge, this is the first study to elucidate the potential in vitro degradability, associated to an extensive morphological analysis, of three natural polymeric biodegradable membranes from heterologous origin, commercially available and CE-certified for oral applications. This work is promising and may help clinicians to select the most suitable GBR membrane for each patient and clinical situation; but also preliminary, and some limitations need to be addressed before completing the total characterization, as determining the potential for osteogenesis, angiogenesis, and osteoimmunomodulation for guide bone regeneration is essential. The contact angle measurement would also provide information on the hydrophilicity and hydrophobicity of the membranes surface. Overall, the present study provides an extensive morphological analysis of three collagen-based membranes with potential for use in regenerative medicine and dentistry, but additional nanostructural characterization through field emission scanning electron microscopy, atomic force microscopy, Raman spectroscopy and nanomechanical characterizations should also be performed in order to analyze how membranes degrade and how these processes may be retarded. Therefore, even when present results represent an important step forward, they may be taken with caution as future in vitro and vivo research strategies are required.

## 5. Conclusions

Derma Fina, the bi-layered porcine types I/III collagen membrane, has shown the highest resistance to tested degradation challenges under in vitro conditions. Duo-Teck, from equine lyophilized collagen felt, was the most susceptible membrane, and sings of complete degradation started to appear as soon as after 8 h of immersion. The collagenase solution from *C. histolyticum* performed as the most aggressive test for the membranes’ degradation studies, as all membranes presented 100% degradation before the end of study. Pores larger than 100 µm appeared during the degradation processes of all tested membranes, which may jeopardize the soft tissue cells barrier effect required for a successful GBR therapy.

## Figures and Tables

**Table 1:** (**a**) Thickness (mm) analysis of the three experimental membranes (Derma Fina, Evolution Standard and Duo-Teck) after immersions periods up to 21 days in PBS (Hydrolytic degradation test), collagenase from *Clostridium histolyticum* (Bacterial collagenase resistance test) and trypsin (Enzyme resistance test). Values shown are mean and standard deviation. (**b**) Attained *p* values after pairwise comparisons between membranes’ thicknesses after the different immersion times and the initial thickness (thickness at t_0_: Th_0_). Significance was considered at *p* ≤ 0.001.

(**a**)									
		**Derma**			**Evolution**			**Duo-Teck**	
	**Trypsin**	***C. hystolyticum***	**PBS**	**Trypsin**	***C. hystolyticum***	**PBS**	**Trypsin**	***C. hystolyticum***	**PBS**
t_0_	1.02 (0.13)	1.09 (0.17)	1.22 (0.04)	0.43 (0.14)	0.39 (0.05)	0.47 (0.01)	0.15 (0.00)	0.15 (0.01)	0.17 (0.00)
4 h	0.75 (0.05)	0.59 (0.05)	0.85 (0.10)	0.21 (0.02)	0.14 (0.02)	0.20 (0.03)	0.13 (0.04)	0.14 (0.01)	0.18 (0.06)
8 h	0.64 (0.07)	0.54 (0.05)	0.75 (0.08)	0.20 (0.06)	0.08 (0.01)	0.23 (0.03)	0.11 (0.03)	0 (0)	0.14 (0.05)
12 h	0.61 (0.06)	0.48 (0.07)	0.66 (0.03)	0.16 (0.04)	0.06 (0.01)	0.21 (0.04)	0.08 (0.01)	0 (0)	0.11 (0.05)
16 h	0.60 (0.05)	0.41 (0.04)	0.66 (0.05)	0.16 (0.04)	0.06 (0.01)	0.20 (0.03)	0.07 (0.02)	0 (0)	0.08 (0.02)
24 h	0.57 (0.02)	0.36 (0.03)	0.63 (0.04)	0.08 (0.02)	0.05 (0.01)	0.16 (0.06)	0.04 (0.04)	0 (0)	0.09 (0.01)
48 h	0.51 (0.04)	0.34 (0.02)	0.63 (0.03)	0.06 (0.03)	0.05 (0.01)	0.13 (0.01)	0.03 (0.03)	0 (0)	0 (0)
72 h	0.49 (0.05)	0.28 (0.03)	0.60 (0.05)	0.03 (0.02)	0.03 (0.01)	0.10 (0.03)	0.01 (0.02)	0 (0)	0 (0)
7 d	0.49 (0.02)	0.20 (0.05)	0.59 (0.03)	0.03 (0.03)	0 (0)	0.05 (0.03)	0.01 (0.01)	0 (0)	0 (0)
14 d	0.53 (0.07)	0.08 (0.04)	0.56 (0.04)	0.04 (0.03)	0 (0)	0.04 (0.02)	0 (0)	0 (0)	0 (0)
21 d	0.57 (0.07)	0 (0)	0.52 (0.08)	0.04 (0.03)	0 (0)	0.05 (0.01)	0 (0)	0 (0)	0 (0)
(**b**)									
Th_0_-Th_1_	0.002	<0.001	<0.001	0.008	<0.001	<0.001	0.152	0.602	0.323
Th_0_-Th_4h_	0.001	<0.001	<0.001	0.002	<0.001	<0.001	0.009	<0.001	0.46
Th_0_- Th_8h_	<0.001	<0.001	<0.001	0.002	<0.001	<0.001	0.02	<0.001	0.052
Th_0_-Th_12h_	<0.001	<0.001	<0.001	0.001	<0.001	<0.001	0.035	<0.001	<0.001
Th_0_-Th_16h_	<0.001	<0.001	<0.001	0.001	<0.001	<0.001	0.001	<0.001	<0.001
Th_0_-Th_24h_	<0.001	<0.001	<0.001	0.001	<0.001	<0.001	<0.001	<0.001	<0.001
Th_0_-Th_48h_	<0.001	<0.001	<0.001	0.001	<0.001	<0.001	<0.001	<0.001	<0.001
Th_0_- Th_72h_	<0.001	<0.001	<0.001	0.001	<0.001	<0.001	<0.001	<0.001	<0.001
Th_0_-Th_7d_	<0.001	<0.001	<0.001	0.001	<0.001	<0.001	<0.001	<0.001	<0.001
Th_0_-Th_14d_	<0.001	<0.001	<0.001	<0.001	<0.001	<0.001	<0.001	<0.001	<0.001
Th_0_-Th_21d_	<0.001	<0.001	<0.001	<0.001	<0.001	<0.001	<0.001	<0.001	<0.001

**Table 2:** (**a**) Weight (µg) analysis of the three experimental membranes (Derma Fina, Evolution Standard and Duo-Teck) after immersion periods up to 21 days in PBS (Hydrolytic degradation test), collagenase from *Clostridium histolyticum* (Bacterial collagenase resistance test) and trypsin (Enzyme resistance test). Values shown are mean and standard deviation. (**b**) Attained *p* values after pairwise comparisons between membranes’ weights after the different immersion times and the initial weight (weight at t_0_: W_0_). Significance was considered at *p* ≤ 0.001.

(**a**)									
		**Derma**			**Evolution**			**Duo-Teck**	
	**Trypsin**	***C. hystolyticum***	**PBS**	**Trypsin**	***C. hystolyticum***	**PBS**	**Trypsin**	***C. hystolyticum***	**PBS**
t_0_	55.47 (4.04)	57.49 (1.54)	59.69 (1.13)	15.52 (3.19)	14.78 (0.70)	16.04 (0.13)	8.83 (0.40)	9.35 (0.52)	8.9 (0.28)
4 h	53.25 (3.71)	55.58 (1.61)	58.62 (1.17)	14.42 (2.96)	13.67 (0.44)	15.89 (0.10)	7.83 (0.35)	6.28 (0.31)	8.70 (0.17)
8 h	53.19 (3.68)	53.02 (0.68)	58.34 (1.33)	14.67 (2.78)	12.30 (0.46)	15.8 (0.04)	7.60 (0.54)	0 (0)	8.34 (0.09)
12 h	51.95 (3.60)	49.32 (0.39)	56.45 (1.11)	14.89 (2.86)	10.94 (0.47)	15.27 (0.12)	7.20 (0.14)	0 (0)	6.53 (1.20)
16 h	52.42 (3.64)	47.90 (0.15)	57.64 (1.24)	14.08 (2.98)	9.04 (0.07)	15.41 (0.08)	3.88 (0.75)	0 (0)	6.92 (0.79)
24 h	52.21 (3.62)	44.87 (0.04)	57.11 (1.47)	14.04 (3.06)	7.79 (0.35)	15.67 (0.07)	1.16 (1.27)	0 (0)	6.47 (0.45)
48 h	53.27 (3.79)	41.93 (0.06)	58.25 (1.78)	14.02 (3.27)	6.33 (0.79)	15.6 (0.17)	1.02 (1.12)	0 (0)	0 (0)
72 h	52.73 (3.60)	34.89 (0.49)	56.66 (1.02)	12.95 (3.45)	3.63 (1.27)	13.58 (0.55)	0.97 (1.06)	0 (0)	0 (0)
7 d	52.96 (3.64)	24.34 (1.00)	56.24 (0.68)	11.87 (3.81)	0 (0)	12.13 (0.13)	0.98 (1.07)	0 (0)	0 (0)
14 d	52.88 (3.69)	5.67 (0.80)	54.33 (1.20)	11.26 (3.23)	0 (0)	11.93 (0.07)	0 (0)	0 (0)	0 (0)
21 d	52.89 (3.70)	0 (0)	51.51 (1.55)	10.99 (2.88)	0 (0)	11.66 (0.06)	0 (0)	0 (0)	0 (0)
(**b**)									
W_0_-W_4h_	<0.001	<0.001	<0.001	<0.001	<0.001	<0.001	<0.001	<0.001	0.01
W_0_-W_8h_	<0.001	<0.001	<0.001	<0.001	<0.001	0.003	<0.001	<0.001	0.044
W_0_-W_12h_	<0.001	<0.001	<0.001	<0.001	<0.001	<0.001	<0.001	<0.001	0.002
W_0_-W_16h_	<0.001	<0.001	<0.001	<0.001	<0.001	<0.001	<0.001	<0.001	<0.001
W_0_-W_24h_	<0.001	<0.001	<0.001	<0.001	<0.001	<0.001	<0.001	<0.001	<0.001
W_0_-W_48h_	<0.001	<0.001	<0.001	<0.001	<0.001	0.015	<0.001	<0.001	<0.001
W_0_-W_72h_	<0.001	<0.001	<0.001	<0.001	<0.001	<0.001	<0.001	<0.001	<0.001
W_0_-W_7d_	<0.001	<0.001	<0.001	<0.001	<0.001	<0.001	<0.001	<0.001	<0.001
W_0_-W_14d_	<0.001	<0.001	<0.001	<0.001	<0.001	<0.001	<0.001	<0.001	<0.001
W_0_-W_21d_	<0.001	<0.001	<0.001	<0.001	<0.001	<0.001	<0.001	<0.001	<0.001
